# Validity and Reliability of the Fatigue Severity Scale in an Adult Swedish Burn Population

**DOI:** 10.3390/ebj7010014

**Published:** 2026-03-02

**Authors:** Sara Enblom, Fredrik Huss

**Affiliations:** 1 Department of Surgical Sciences, Plastic Surgery, Uppsala University, 751 85 Uppsala, Sweden; fredrik.huss@uu.se; 2 Burn Center, Department of Plastic and Maxillofacial Surgery, Uppsala University Hospital, 751 85 Uppsala, Sweden

**Keywords:** burns, psychometrics, quality of life, activity performance, depression, anxiety

## Abstract

**Highlights:**

**What are the main findings?**

1. The Fatigue Severity Scale (FSS) demonstrates a high concurrent validity compared to the Brief Fatigue Inventory (BFI).
2. A high internal consistency was found (α > 0.9) for all items in both FSS and BFI.

**What are the implications of the main findings?**

1. The FSS will be a useful tool in connection with clinical follow-ups at the Burn Center in Uppsala.

**Abstract:**

**Background**: A burn injury is a complex trauma often followed by complications, one of which is fatigue. The objective of this study was to validate the Fatigue Severity Scale (FSS) in an adult Swedish burn cohort. **Methods**: A prospective cohort study was performed at the Burn Center at Uppsala University Hospital in Uppsala, Sweden. All patients who were registered for follow-up 6 months after their burn injury were asked to participate. Included patients completed questionnaires at 6 and 12 months postburn. Psychometric properties were investigated, including internal consistency (Cronbach’s alpha) and concurrent validity, comparing FSS with the Brief Fatigue Inventory (BFI), which was considered to be the “gold standard.” Convergent validity was investigated among the fatigue assessments and quality of life, depression/anxiety, and daily activities. **Results**: In total, 70 included patients attended both visits. FSS demonstrated high internal consistency (Cronbach’s alpha: 0.96 at both timepoints). There was high concurrent validity between FSS and BFI on both occasions (Spearman’s rho: 0.816 and 0.863, respectively), and the convergent validity was strong. **Conclusions**: The result indicates that the two fatigue scales correspond well to each other and that the FSS, therefore, is a valid and reliable assessment of fatigue in adult Swedish burn patients.

## 1. Introduction

A burn is a complex traumatic injury that often needs multidisciplinary care and rehabilitation [1]. Common sequelae are pain, muscle weakness, and scar contractures [2]. Another complication is fatigue [3,4], which is a symptom that is seldom measured.

No generally accepted definition of fatigue exists [5], and many agree that it is difficult to define, being complex and multidimensional [5,6]. However, definitions often agree that fatigue is overwhelming, and it is described as a feeling of tiredness, exhaustion, weakness, and lack of energy. These definitions are all subjective and should be measured with self-reported methods [5,6,7,8].

A patient-reported outcome (PRO) gives information about the patient’s health status, which is reported by the patients themselves, without the influence of a healthcare professional or anyone else [9]. PROs are essential in those cases where it is difficult to externally observe outcomes that are important to the patient and sometimes the only way to evaluate treatment of conditions such as pain and fatigue [10]. PROs also represent the patient’s view of how the condition and/or the treatment affects everyday functioning and well-being [11]. A PRO can be measured by self-report or by interview with a clinician [9], stressing the importance of self-assessment and subjective measures. There are specific and standardized fatigue assessment tools, for example, the Fatigue Severity Scale (FSS) and the Brief Fatigue Inventory (BFI) [12]. Many of the scales are, or claim to be, disease-specific or have only been applied to a few diseases. The BFI has been, for example, validated for burn patients in Australia [4].

To our knowledge, there is no specific fatigue measurement tool for burn patients in Sweden. At the Burn Center at Uppsala University Hospital, the FSS has been used for some years, since it has been translated into Swedish and validated for other diagnoses in Sweden [13]. Even if the psychometric properties of the FSS were previously assessed in several patient groups, Friedman et al. [14] consider it important to assess this specifically in the actual patient group since cultural differences could also influence the results [15,16].

The aim of the present study was to evaluate the psychometric properties of the Swedish version of the FSS and validate it in an adult Swedish burn population.

The hypothesis was that the two fatigue measures, FSS and BFI, would demonstrate strong convergent validity and be highly correlated.

## 2. Materials and Methods

### 2.1. Participants and Data Collection

A prospective cohort study was performed at Uppsala University Hospital, Sweden, between May 2020 and December 2024. All patients scheduled for follow-up—according to clinic’s routine: 6 ± 3 and 12 ± 3 months, at the Burn Center’s outpatient clinic in Uppsala—are routinely asked to fill out several questionnaires with the purpose of screening any problems that might have arisen following the burn injury. Patients registered for follow-up 6 months after burn injury were informed about the study and asked to participate. Oral and written information were given prior to the consent. The inclusion criteria were burn patients ≥ 18 years, admitted < 24 h post trauma, and understands written and spoken Swedish. Exclusion criteria were any underlying condition that made it impossible to complete the questionnaires (e.g., dementia or other psychiatric condition) and the patient choosing not to attend the routine 12-month follow-up.

Due to the relatively small population of burn patients in Sweden, it was decided to deviate from the common rule of thumb that suggests 5–10 participants per item. Therefore, 70 patients with data from both follow-up visits were set according to the power analysis made in SPSS for Windows (version 23, IBM Corp, Armonk, NY, USA). The number of patients recommended was 67. To reduce bias as much as possible, patients with missing data from more than one questionnaire were excluded [17,18].

The patients were asked to fill out the questionnaires in connection with the visit. The completion of the questionnaires was estimated to take 30 minutes during each visit.

Apart from the ordinary fatigue assessment scale (FSS), which all patients fill out, the study patients included were also asked to fill out the BFI at both visits.

Some of the patients received treatment between the follow-up visits, either at the Burn Center’s outpatient clinic or at the local hospital. The treatment most often consisted of scar treatment or stretching exercises.

### 2.2. Outcome Measures

Fatigue Severity Scale (FSS) is a unidimensional self-assessment scale [19] that measures the severity of fatigue and its impact on daily activities, motivation, work, and relationships (File S1) [20]. The scale includes nine statements on a seven-point Likert rating scale, ranging from 1 (“completely disagree”) to 7 (“completely agree”) [21]. The FSS cutoff score for fatigue is set to ≥4, where higher scores indicate greater fatigue severity [21,22,23,24]. The Swedish version of FSS has shown good psychometric properties when tested on patients with systemic lupus erythematosus (SLE) [13].

The BFI is, like the FSS, a unidimensional [6] self-assessment scale (File S1). BFI is a valid and reliable tool for measuring fatigue in burn patients in Australia during the first 12 months after burn [4]. BFI consists of nine questions and assesses the impact that fatigue has on daily activities, mood, work, and social relations. The questions are rated on an 11-point numeric rating scale, from 0 = no impact to 10 = very serious impact [4]. The average of the nine items indicates the degree of fatigue. A higher mean corresponds to a higher degree of fatigue [4]. The original study by Mendoza et al. [25] presents the following cutoff points: mild (1–3), moderate (4–6), and severe (7–10). The BFI is translated into Swedish but has not been validated in a Swedish patient cohort.

EuroQol 5-Dimension 3-Level version (EQ-5D-3L) is a standardized self-assessment tool for measuring health-related quality of life and current self-reported health state [26]. Part one contains five dimensions: mobility, self-care, usual activities, pain/discomfort, and anxiety/depression. Part two, EQ VAS, is an assessment of the current state of health using a vertical visual analogue scale, divided into 100 steps, where 0 is labelled “the worst health you can imagine” and 100 “the best health you can imagine.” EQ-5D shows a high clinical validity and good psychometric values in Swedish burn patients [27]. In this study, only the EQ VAS was used [26,28].

The Hospital Anxiety and Depression Scale (HADS) was used in this study since the symptoms of fatigue and depression often parallel each other and sometimes overlap [3]. It is a validated self-assessment scale and consists of 14 statements, 7 about anxiety and 7 about depression, which are computed separately [29]. Each statement has four answers to choose from (0–3). The possible scores range from 0 to 21 for anxiety and 0 to 21 for depression. A score of 0 to 7 for either subscale is regarded as being in the normal range, 8–10 suggests the presence of either state, and a score of 11 or higher indicates the probable presence of the respective state [30]. The Swedish version of HADS was proven useful as a clinical indicator of depression and anxiety [31].

The Patient and Observer Scar Assessment Scale (POSAS) is a subjective estimation of the scars. It contains two scales: the patient scale and the observer scale. Only the patient scale was used in the present study. The patient scale consists of six items, which describe the scar in terms of pain, itch, colour, stiffness, thickness, and irregularity. A seventh item includes giving an overall opinion of the scar, ranging from 1 to 10; a higher score indicates a more severe scar. The POSAS is valid and reliable for burn scars [32] and has been translated into Swedish. To our knowledge, the POSAS has not been validated in a Swedish-speaking population. In this study, we included the score from the overall question: “What is your overall opinion of the scar compared to normal skin?”

Performance and Satisfaction in Activities of Daily Living (PS-ADL) is a self-assessment tool in which the patient assesses 39 everyday activities, divided into 12 dimensions, and the level of difficulty with which each activity is performed. The mean sum of the 12 dimensions is calculated and used as the final score (0–3). The higher the score, the more problems with daily activities. PS-ADL is valid and reliable for Swedish patients with rheumatoid arthritis, according to Archenholz and Dellhag [33], and is commonly used in research and clinical settings.

### 2.3. Statistical Analyses

Data were analyzed using IBM SPSS Statistics for Windows (version 23, IBM Corp, Armonk, NY, USA). Psychometric properties of the included outcome measures were evaluated [34]. The correlations were made using Spearman’s rho correlation analysis. The threshold for statistical significance was *p* < 0.05. A linear regression analysis was also performed to quantify the strength of the relationship between variables. An R^2^ value of 0.6–0.8 was considered a strong correlation.

### 2.4. Reliability

A measurement with high reliability gives stable, reproducible, and precise measures at different time points. Internal consistency (Cronbach’s alpha) is the correlation between each item and between each item and the total FSS and BFI scores, respectively. It was calculated at 6 and 12 months in each scale, with a recommended alpha > 0.9, desirable for clinical applications [4,35,36]. The two fatigue scales differ somewhat from each other regarding statements/questions, different scale steps, and different cutoffs, but since only the total scores of both scales were used in the statistical analysis, the scores were considered equivalent. Another form of reliability is the test-retest reliability, which captures the stability of the measurement using repeated assessments [37]. However, this test was not performed within the scope of this study, but uses the test-retest for FSS already made by Krupp et al. [21]. No test-retest was performed for the BFI when developing the measurement in 1999 by Mendoza et al. [25].

The standard error of measurement (SEM) represents random and unsystematic fluctuations that are due to chance [38]. If the difference between two measurements is more than the SEM value, it may indicate that the change is real and true. The smaller the SEM, the more reliable the measurements [39].

The minimal detectable change (MDC) is a statistical estimate of the smallest amount of change that can be detected by a measure and is consistent with a notable change in capacity but is not the result of a measurement error [40].

### 2.5. Validity

Criterion validity contains the subgroups’ concurrent and predictive validity. Concurrent validity was used to measure the correlation with another fatigue instrument (“gold standard”), measured at the same timepoints, in this case, BFI [34]. The concurrent validity was computed using Spearman’s rho. Results were considered to be statistically significant when *p* < 0.05. Predictive validity measures how well an instrument can predict future results and how much of the variation of the BFI is explained by the FSS. This was measured in BFI and FSS, at both timepoints, using a linear regression model [41].

Construct validity contains the subgroups’ convergent and content validity and aims to verify how well a test measures the concept it was designed to evaluate. The construct validity of the BFI was established by Toh et al. [4]. Convergent validity of FSS and BFI was evaluated in this study through the correlation with other outcome measures, e.g., EQ VAS, HADS, and PS-ADL, and computed using Spearman’s rho [42]. Results were considered significant when *p* < 0.05. Also, a linear regression analysis was computed to investigate the strength of the correlation between the measures.

Content validity of the FSS was predetermined from the Swedish version of FSS [13], but in another disease group (systemic lupus erythematosus, SLE). Content validity has not been established in this current cohort since it requires a qualitative study design, which was not feasible within the scope of this study [13].

### 2.6. Floor and Ceiling Effects

Floor and ceiling effects were calculated for both FSS and BFI at both timepoints. Floor and ceiling effects are present if more than 15% of the respondents score the lowest or highest possible score [43,44], thus indicating that the measurement tool does not have a sufficient span to capture differences.

## 3. Results

### 3.1. Participants and Characteristics

In total, 169 adult patients who came for follow-up 6 months (±3 months) postburn from May 2020 to June 2024 were screened for inclusion. In total, 60 patients could not be included due to violation of inclusion/exclusion criteria, e.g., psychiatric condition (dementia), not being able to manage spoken or written Swedish, late follow-up, etc., or missed inclusions.

In total, 24 patients declined to participate, leaving 85 patients in the study.

**Figure 1:**
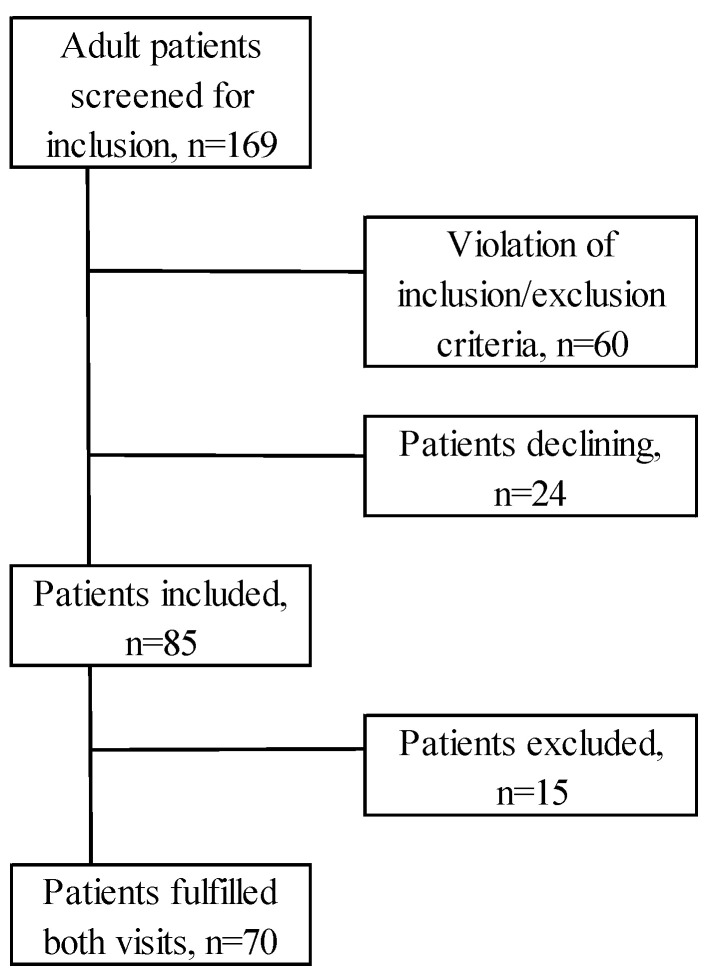
Inclusion and exclusion.

Most of the questionnaires were completed during the follow-up visits. In all, 15 patients (17.6%) were excluded due to failure to appear at the 12-month visit, late visit, or missing data/incomplete questionnaires (Figure 1). There were no notable differences in the characteristics of those excluded compared to the patients included.

The recruitment of participants was, in 92% of the cases, done by the first researcher (SE), who was also one of their treating health practitioners.

In total, 70 patients fulfilled both follow-up visits by December 2024 (Table 1), of these 84% underwent surgery as a result of their burn. Aside from three respondents lacking the POSAS 12-month questionnaire, all the questionnaires were filled out completely, except for one respondent whose data were missing for one question on the BFI. Mean imputation was used by replacing this score with the mean value of the other eight questions for this patient.

### 3.2. Reliability

The internal consistency is presented in Table 2. Cronbach’s alpha coefficient was >0.9 for all items in both FSS and BFI, at both timepoints, even when items were deleted, indicating a high internal consistency according to Toh et al. [4].

FSS had the best estimates of reliability and precision (SEM), with a score of 0.40 at 6 months and 0.36 at 12 months, indicating that a change of less than 0.40/0.36 may be due to measurement error. The corresponding values for the BFI were 0.54/0.57 (Table 3).

The results of the minimal detectable change (MDC) showed that the FSS requires less change of score than the BFI for the patients to experience a real difference between measurement timepoints (6 months) (Table 3).

### 3.3. Validity

**Figure 2:**
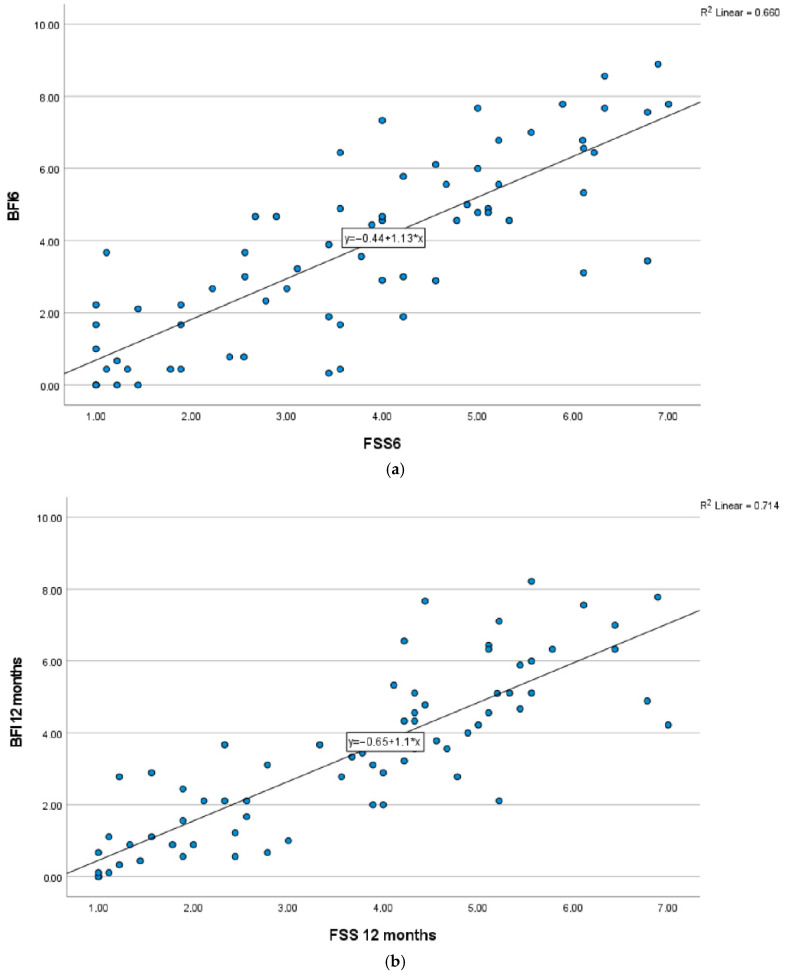
(**a**) Linear regression 6 months, dependent variable: BFI, independent variable: FSS. (**b**) Linear regression 12 months, dependent variable: BFI, independent variable: FSS.

The Spearman’s rho correlation between FSS and BFI was high on both occasions, 0.816 and 0.863, respectively (*p* < 0.001), demonstrating a high concurrent validity. The linear regression analysis, used to establish the predictive validity, also showed a relatively strong correlation, R^2^ = 0.66 at 6 months and 0.71 at 12 months, which means that 66% of the BFI score is explained by FSS at 6 months and 71% at 12 months (Figure 2a,b).

The convergent validity of FSS and BFI showed a similar pattern to other outcome measures. A high statistically significant correlation was seen between the fatigue scales and EQ-VAS (negative), HADS anxiety and depression, and PS-ADL (*p* < 0.001). A non-significant, or weak, correlation was found with TBSA% and varying results from the correlation with POSAS. The regression analysis revealed low (R^2^ < 0.4) degrees of explanation for all variables except HADS anxiety and depression (12 months FSS; 6 and 12 months BFI) and EQ VAS (12 months FSS), which had moderate degrees. The differences in value between FSS and BFI are noteworthy in some of the variables, where EQ VAS (12 months), HADS anxiety (6 months), and HADS depression (6 months) exceed a difference of 0.1 (Table 4).

Neither of the fatigue scales showed any floor or ceiling effects that needed to be considered [43] (Table 5).

## 4. Discussion

The primary aim of the study was to establish if the Fatigue Severity Scale (FSS) is reliable and valid in an adult Swedish burn cohort.

The results show a strong association between the overall scores from the FSS and the BFI, which implies that the FSS is a reliable and valid measurement tool and equivalent to the BFI. This is consistent with previous research indicating that the FSS provides acceptable reliability and precision [22].

### 4.1. Study Design

The design of the study used the two fatigue scales in parallel and at the same timepoints to compare them under the same conditions. With an intention to limit the amount of missing data in the study, the patients were offered the opportunity to ask the staff at the time of the visit if they had difficulties in completing the questionnaires. A way of minimizing the dropout rate was to contact included patients who did not show up for the 12-month visit and ask them to fill out the questionnaires and return them by mail. The fact that one single person recruited most of the patients might also have played a role in the continuity of the research and the results in terms of inclusion.

### 4.2. Validity and Reliability

Although the psychometric properties of the FSS have been evaluated earlier in other patient groups, fatigue is also important to assess in persons who have suffered a burn injury [45]. High internal consistency (Cronbach’s alpha) was obtained (>0.9). This is in line with studies validating the FSS in other patient groups [13,15,45,46,47,48] and comparable to the result of the BFI in this study.

The standard error of measurement (SEM) shows that the differences in scores between timepoints are small and further strengthens the reliability and accuracy of both scales [39]. Another strength is the result of the minimal detectable change (MDC), where both outcome measures present similar and stable values at both timepoints, which may suggest reliable measurement tools. The fact that, to be considered a notable change, FSS requires less variability in the scores than the BFI might be due to the different rating scales, where FSS has a 7-point scale and the BFI on an 11-point scale.

The concurrent validity is shown by the strong association between the overall scores of the BFI and the FSS, which indicates that they measure similar aspects of fatigue. To show not only the statistically significant correlation (Spearman’s rho), a linear regression analysis was also performed to investigate the relationship between the two fatigue scales. The linear regression analysis shows a high predictive validity, meaning that a high percentage of the variation can be explained, or predicted, by the FSS.

Statistically significant correlations (Spearman’s rho) are found between both fatigue scales and quality of life, psychological outcomes, and the performance of daily activities, establishing convergent validity. Both measurement tools indicate that the more severe the fatigue, the more severe the depressive and anxiety symptoms, and the greater the impairment of quality of life and the performance of daily activities. As far as the FSS is concerned, a similar result was found in the study by Rossi et al. [23]. Moderate to strong correlations have also been found between the FSS and the assessment of fatigue using the visual analogue scale (VAS) [21,24] and with scales measuring depression [21,22,23] and quality of life [15,23]. In this current study, nonexistent or weak correlations were found for both fatigue scales regarding POSAS and TBSA%. The latter may be somewhat intuitively surprising, but it presents another similarity to suggest that the two scales measure in a similar way in contrast to previous research, which found strong correlations between these variables [3,49,50].

Linear regression analysis reveals a low to moderate degree of explanation for all outcome measures except between the FSS and the BFI. The low degrees imply that the independent variables do not explain the variation of fatigue to a great extent. In turn, this might be because fatigue is a complex and multifactorial experience [51] that is not fully captured by the questions in the other assessments and therefore does not reliably predict fatigue. Still, the conformity between both fatigue scales is high, showing similar values for all variables, except EQ VAS (12 months), HADS anxiety (6 months), and HADS depression (6 months), where the difference between the fatigue measurement tools lies between 0.1 and 0.3. This might indicate that they measure fatigue in slightly different ways and may be of varying clinical utility.

The floor and ceiling effects are small for FSS in this study, which is in line with previous studies [13,15,46,47].

### 4.3. Missing Data

Respondents with more than one missing outcome measure were excluded from the analysis. This might lead to biased results, unless the assumption is that data are missing completely at random (MCAR), as in this study, meaning that the subjects with missing outcome measures are a random subset of the complete sample [17]. The high completion rate of each outcome measure minimizes bias caused by missing data. The fact that three of the respondents included have one missing outcome measure each, POSAS 12 months, is not expected to affect the final result.

### 4.4. Strengths and Limitations

One strength of this study is that it contains a comparably large sample of this overall small patient group. All patients scheduled for follow-up at the outpatient clinic at Uppsala Burn Center were asked to respond consecutively; thus, the risk of selection bias was low. Another strength is the low number of missing data because patients in most cases filled out the questionnaires during the follow-up visits, and any omissions could be noticed and addressed on the spot. If this was not possible, incomplete questionnaires were sent home to the patients with a request for them to be completed and returned. It is assumed that confounders, if any, should have affected both fatigue scales in the same way, since they were administered at the same point in time.

Loss to follow-up was 17.6% in this study, which lies within the limits of an acceptable loss according to Dumville et al. [52], who stated that a loss greater than 20% means that there is a possible risk of bias.

However, there are also limitations. All outcome measures are self-administered by the participants; therefore, misunderstandings regarding questions and statements cannot be ruled out, even though they have the opportunity to ask the staff, should something be unclear.

The differences between the two fatigue scales have been discussed in light of whether they might have affected the results, for example, statements vs. questions, different scale steps, and different cutoffs. Since the total scores of both scales were used in the statistical analyses and the results corresponded, a conformity was considered to exist.

### 4.5. Implementation

Based on the results of this study, the FSS will be a useful tool in connection with clinical follow-ups at the Burn Center in Uppsala.

## 5. Conclusions

The current study provides novel data on the reliability and validity of the FSS, indicating that the FSS is a valid and reliable measurement tool to assess fatigue in adult burn patients in Sweden.

Future research is needed, however, to establish the content validity of the FSS within the Swedish cohort of burn patients specifically. A test-retest analysis to further strengthen reliability will also be useful.

## Figures and Tables

**Table 1:** Participant characteristics.

Variable	*n* = 70
Gender, m/f, *n* (%)	47 (67.1)/23 (32.9)
Age, yrs median (range) IQR	49 (17–83) 37
Length of stay, days, mean (range) SD	12 (1–48) 11
TBSA %, median (range) IQR	8.0 (1.0–37.0) 11.25
FSS ≥ 4, *n* total (%) m/f	33 (47.1) 24/9

TBSA—total body surface area burned; FSS—Fatigue Severity Scale.

**Table 2:** Internal consistency of the FSS and BFI, Cronbach’s alpha (α), *n* = 70.

	FSS				BFI			
	6 Months		12 Months		6 Months		12 Months	
Items	Mean (SD)	Cronbach’s Alpha ^a^	Mean (SD)	Cronbach’s Alpha ^a^	Mean (SD)	Cronbach’s Alpha ^a^	Mean (SD)	Cronbach’s Alpha ^a^
1	4.47 (2.06)	0.956	4.51 (2.03)	0.957	4.09 (2.82)	0.946	3.66 (2.70)	0.928
2	2.81 (1.87)	0.963	2.63 (1.79)	0.960	4.07 (2.61)	0.944	3.90 (2.44)	0.924
3	4.19 (2.09)	0.953	3.94 (2.05)	0.948	5.40 (3.00)	0.951	4.97 (2.85)	0.929
4	4.31 (2.10)	0.956	4.14 (2.08)	0.950	3.77 (2.75)	0.944	3.50 (2.86)	0.922
5	3.36 (2.01)	0.954	3.27 (1.85)	0.950	3.93 (3.04)	0.945	3.30 (2.88)	0.928
6	3.77 (2.17)	0.955	3.70 (2.04)	0.951	2.20 (2.82)	0.955	1.80 (2.49)	0.943
7	3.51 (1.92	0.955	3.70 (1.99)	0.951	3.39 (3.08)	0.945	3.40 (2.98)	0.930
8	3.57 (2.24)	0.955	3.47 (2.20)	0.953	3.36 (3.06)	9.952	2.63 (2.75)	0.927
9	3.50 (2.14)	0.952	3.64 (2.14)	0.949	3.66 (3.22)	0.944	3.40 (3.00)	0.930
Total	3.72 (1.80)	0.960	3.67 (1.74)	0.957	3.76 (2.50)	0.953	3.40 (2.27)	0.937

^a^ = if items deleted.

**Table 3:** FSS and BFI; mean, confidence interval, standard error of measurement, and minimal detectable change, *n* = 70.

	FSS	BFI
6 months, mean (SD)	3.72 (1.80)	3.76 (2.50)
Confidence Interval (CI)	3.29–4.15	3.16–4.36
Standard Error of Measurement (SEM)	0.40	0.54
Minimal Detectable Change (MDC)	1.12	1.50
12 months, mean (SD)	3.67 (1.74)	3.40 (2.27)
Confidence Interval (CI)	3.25–4.08	2.84–3.92
Standard Error of Measurement (SEM)	0.36	0.57
Minimal Detectable Change (MDC)	1.02	1.58

**Table 4:** Convergent validity of the FSS and BFI, *n* = 70 ^a^.

Variables/Assessments	FSS				BFI			
	6 Months		12 Months		6 Months		12 Months	
	r_s_	R^2^	r_s_	R^2^	r_s_	R^2^	r_s_	R^2^
EQ VAS	−0.57 ***	0.28	−0.68 ***	0.48	−0.51 ***	0.27	−0.69 ***	0.18
POSAS	0.35 **	0.11	0.22 ns	0.06	0.39 ***	0.14	0.26 *	0.06
HADS anxiety	0.56 ***	0.32	0.67 ***	0.42	0.66 ***	0.44	0.68 ***	0.46
HADS depression	0.62 ***	0.36	0.74 ***	0.52	0.76 ***	0.58	0.72 ***	0.47
TBSA%	0.52 ns	0.00	−0.15 ns	0.01	−0.10 ns	0.00	−0.28 *	0.04
PS-ADL	0.51 ***	0.19	0.44 ***	0.14	0.41 ***	0.13	0.45 ***	0.16

^a^—POSAS 12 months, *n* = 67; r_s_—Spearman’s rho, rank correlation coefficient; * correlation is statistically significant at the 0.05 level; ** correlation is statistically significant at the 0.01 level; *** correlation is statistically significant at the 0.001 level; ns—non-significant; R^2^—linear regression; EQ VAS—EuroQol 5D-3L instrument, VAS—visual analogue scale; POSAS—Patient and Observer Scar Assessment Scale, total score, patient scale; HADS—Hospital Anxiety and Depression Scale; TBSA%—total body surface area %, burned; PS-ADL—Performance and Satisfaction in Activities of Daily Living.

**Table 5:** Floor and ceiling effect of FSS and BFI, based on total scores, *n* = 70.

	FSS		BFI	
	Floor	Ceiling	Floor	Ceiling
6 months, *n*/%	6/8.6	1/1.4	5/7.1	0/0.0
12 months, *n*/%	5/7.1	7/10.0	0/0.0	0/0.0

FSS—Fatigue Severity Scale; BFI—Brief Fatigue Inventory.

## Data Availability

The raw data supporting the conclusions of this article will be made available by the authors on request.

## Supplementary Materials

The following supporting information can be downloaded at: https://www.mdpi.com/article/10.3390/ebj7010014/s1, File S1: Kort Formulär Om Trötthet.

## Abbreviations

The following abbreviations are used in this manuscript:

BFI	Brief Fatigue Inventory
CI	confidence interval
EQ-5D-3L	EuroQol 5-Dimension 3-Level version
EQ VAS	EuroQol visual analogue scale
FSS	Fatigue Severity Scale
HADS	Hospital Anxiety and Depression Scale
MCAR	missing completely at random
MDC	minimal detectable change
POSAS	Patient and Observer Scar Assessment Scale
PRO	patient-reported outcome
PS-ADL	Performance and Satisfaction in Activities of Daily Living
SD	standard deviation
SEM	standard error of measurement
TBSA	total body surface area
