# Supplementary material for: Validity and Reliability of the Fatigue Severity Scale in an Adult Swedish Burn Population
Source: Eur Burn J. 2026 Mar 2;7(1):14. doi: 10.3390/ebj7010014 (PMC13025754; doi:10.3390/ebj7010014)
Supplement: Supplementary file 1 [file ebj-07-00014-s001.zip › ebj-4114864-supplementary.pdf]

Kort Formulär Om Trötthet

ID-NR I STUDIEN: ____________ SJUKHUS NR: ____________

Dagens datum: ______/ ______/ ______ Tid: ________________

Namn: ____________________ ______________________ ____________________

Efternamn Förnamn Andra förnamn

De flesta människor har någon gång i livet känt sig väldigt trötta eller utmattade Har du känt dig ovanligt trött eller utmattad den senaste veckan? Ja ☐ Nej ☐

1. Gradera din trötthet (slitenhet, utmattning) genom att sätta en ring runt den siffra som bäst beskriver hur trött du känner dig just NU.

0 1 2 3 4 5 6 7 8 9 10

Ingen trötthet — Värsta tänkbara trötthet

2. Gradera din trötthet (slitenhet, utmattning) genom att sätta en ring runt den siffra som bäst beskriver hur trött du NORMALT har varit under det senaste dygnet.

0 1 2 3 4 5 6 7 8 9 10

Ingen trötthet — Värsta tänkbara trötthet

3. Gradera din trötthet (slitenhet, utmattning) genom att sätta en ring runt den siffra som bäst beskriver hur trött du varit som VÄRST under det senaste dygnet.

0 1 2 3 4 5 6 7 8 9 10

Ingen trötthet — Värsta tänkbara trötthet

4. Sätt en ring runt den siffra som bäst beskriver hur din trötthet under det senaste dygnet har inverkat på:

A. Dina allmänna aktiviteter

0 1 2 3 4 5 6 7 8 9 10

Ingen inverkan — Mycket stor inverkan

B. Ditt humör

0 1 2 3 4 5 6 7 8 9 10

Ingen inverkan — Mycket stor inverkan

C. Din gångförmåga

0 1 2 3 4 5 6 7 8 9 10

Ingen inverkan — Mycket stor inverkan

D. Det arbete du normalt utför (både arbete utanför hemmet och hushållssysslor)

0 1 2 3 4 5 6 7 8 9 10

Ingen inverkan — Mycket stor inverkan

E. Dina relationer till andra människor

0 1 2 3 4 5 6 7 8 9 10

Ingen inverkan — Mycket stor inverkan

F. Din förmåga att njuta av livet

0 1 2 3 4 5 6 7 8 9 10

Ingen inverkan — Mycket stor inverkan

Copyright 1999
The University of Texas M. D. Anderson Cancer Center
All rights reserved.

2001-02-05

Namn:____ Datum:__________________

Trötthetsskala
Fatigue severity scale, FSS (Krupp et al 1989)

Läs igenom följande påståenden och ange i vilken grad Du instämmer i vart och ett genom att ringa in en siffra. Val av siffran 1 innebär att Du inte alls instämmer med påståendet och siffran 7 att Du instämmer helt och hållet. Du kan välja vilken siffra som helst däremellan.

1. Min motivation försämras när jag är trött

1	2	3	4	5	6	7
instämmer inte alls						instämmer helt och hållet

2. Min trötthet försämras av motion

1	2	3	4	5	6	7
instämmer inte alls						instämmer helt och hållet

3. Jag blir lätt trött

1	2	3	4	5	6	7
instämmer inte alls						instämmer helt och hållet

4. Trötthet påverkar min fysiska aktivitet negativt

1	2	3	4	5	6	7
instämmer inte alls						instämmer helt och hållet

5. Trötthet orsakar ofta problem för mig

1	2	3	4	5	6	7
instämmer inte alls						instämmer helt och hållet

6. Trötthet hindrar mig från att utföra en långvarig fysisk aktivitet

1<br>instämmer<br>inte alls	2	3	4	5	6	7<br>instämmer<br>helt och hållet

7. Trötthet påverkar utförandet av vissa plikter och åtaganden negativt

1<br>instämmer<br>inte alls	2	3	4	5	6	7<br>instämmer<br>helt och hållet

8. Trötthet är ett av mina tre mest handikappande symtom

1<br>instämmer<br>inte alls	2	3	4	5	6	7<br>instämmer<br>helt och hållet

9. Trötthet påverkar mitt arbete, mitt familjeliv eller sociala liv negativt

1<br>instämmer<br>inte alls	2	3	4	5	6	7<br>instämmer<br>helt och hållet

Fatigue Severity scale, FSS (uppdaterad 2006-09-05)

Mätområde	Målgrupp	Typ av mätmetod	Tidsåtgång
KROPPSFUNKTION/ KROPPSSTRUKTUR AKTIVITET/ DELAKTIGHET Svårigheter till följd av trötthet	Personer med systemisk lupus erythematosus (SLE) och multipel skleros (MS) samt andra sjukdomar och sjukdomstillstånd	Frågeformulär. Beskrivningsinstrument och testat för utvärdering	Ca 5-10 minuter inkluderande beräkning av poäng
**Utformning**	**Reliabilitet**	**Validitet**	**Kommentarer**
Nio frågor rörande konsekvenser av trötthet med svarsalternativ från 1-7 poäng (likert-skala).	Testat vad gäller "Internal consistency" (samband ingående frågor) och test-retest reliabilitet (stabilitet) över tid på patienter med SLE och MS	Testat vad gäller innehålls-, begrepps- och samtidig validitet, diskriminant validitet (sensitivitet/specificitet) samt "sensitivity"/ känslighet för förändring på patienter med SLE och MS. Formuläret kan skilja ut trötthet vid SLE från trötthet hos friska kontroller med en brytpunkt ("cut-off") vid 4 poäng.	Formuläret är utvecklat i USA och översatt till svenska. Reliabilitet och validitet av den engelska versionen samt två svenska versioner (12) med fokus på patienter med SLE presenteras här. Kontakta carina.bostrom@ki.se för ytterligare information.

Referenser

1. Krupp LB et al. The fatigue severity scale. Application to patients with multiple sclerosis and systemic lupus erythematosus. Arch Neurol 1989; 46: 1121-1123
2. Krupp LB et al. A study of fatigue in systemic lupus erythematosus. J Rheumatol 1990; 17: 1450-1452
3. Schwartz JE et al. The measurement of fatigue: a new instrument. Journal of Psychosomatic Research 1993; 37; 753-762
4. Gladman DD et al. A comparison of five health status instruments in patients with systemic lupus erythematosus (SLE). Lupus 1996; 5; 190-95
5. Austin JS et al. Health outcome improvements in patients with systemic lupus erythematosus using two telephone counselling interventions. Arthritis Care Res 1996; 9: 391-9
6. Ramsey-Goldman R et al. A pilot study on the effects of exercise in patients with systemic lupus erythematosus. Arthritis Care Res 2000,13: 262-9
7. Tench CM et al. The prevalence and associations of fatigue in systemic lupus erythematosus. Rheumatology 2000; 39: 1249-1254
8. Zonana-Nacach A et al. Systemic lupus erythematosus in three ethnic groups.VI: Factors associated with fatigue within 5 years of criteria diagnosis. LUMINA Study Group. Lupus in minority populations: Nature vs Nurture. Lupus 2000; 9: 101-9
9. Tench C et al. Aerobic fitness, fatigue, and physical disability in systemic lupus erythematosus. J Rheumatol 2002; 29; 474-81
10. Omdal R et al. Fatigue in patients with systemic lupus erythematosus: the psychosocial aspects. J Rheumatol 2003; 30: 283-7
11. Neuberger GB. Measures of fatigue. Arthritis & Rheumatism (Arthritis Care & Research) 2003; 49: 175-183
12. Mattsson M. Reliabilitet och validitet av två svenska versioner av Fatigue severity scale vid mätning av trötthet hos patienter med systemisk lupus erythematosus. D-uppsats, Avdelning för sjukgymnastik, Institutionen för hälsovetenskap, Luleå tekniska universitet, 2006

Utrustning	Instruktion till sjukgymnast
Frågeformulär se Protokoll.	Om definition av trötthet enligt ovan används läs då upp den för patienten. Detsamma gäller för tidsangivelse.
**Instruktion till patient**	**Manual och protokoll**
I originalversionen av FSS finns ingen definition av trötthet. I en vidareutveckling av FSS har Schwartz et al (3) utgått från följande definition:<br>"By fatigue we mean a sense of tiredness, lack of energy and total body give-out"<br>("...en känsla av trötthet, kraftlöshet och total kroppslig orkeslöshet")<br><br>Tidsangivelse anges inte i originalversionen av FSS. Tidsangivelsen enligt Schwartz et al (3) är de två senaste veckorna.	protokoll.Fatigue. Severity.scale.pdf

Poängbedömning

Varje fråga ger mellan 1-7 poäng. Ju högre poäng desto svårare trötthet.

Summera svarspoängen. Med nio frågor blir totalsumman 9- 63 poäng.
Totalsumman delas sedan med antal frågor vilket ger en totalpoäng på mellan 1-7 poäng.

Referensdata för friska vuxna finns presenterad (se referenser).
